# *MdSWEET23*, a sucrose transporter from apple (*Malus × domestica* Borkh.), influences sugar metabolism and enhances cold tolerance in tomato

**DOI:** 10.3389/fpls.2023.1266194

**Published:** 2023-10-03

**Authors:** Peixian Nie, Laiping Wang, Miao Li, Deguo Lyu, Sijun Qin, Xiaomin Xue

**Affiliations:** ^1^ Shandong Institute of Pomology, Taian, China; ^2^ College of Horticulture, Shenyang Agricultural University, Shenyang, China

**Keywords:** *MdSWEET23*, apple fruit, sucrose transporter, plasma membrane, cold stress

## Abstract

Photosynthetic products in most fleshy fruits are unloaded via the apoplasmic pathway. Sugar transporters play an important role in the apoplasmic unloading pathway and are involved in sugar transport for fruit development. The *MdSWEET23*, cloned from ‘‘Hanfu’’ apple (*Malus × domestica* Borkh.) fruits, belongs to Clade III of the SWEET family. Subcellular localization revealed that MdSWEET23 is localized on the plasma membrane. β-glucuronidase activity assays showed that *MdSWEET23* was primarily expressed in the sepal and carpel vascular bundle of apple fruits. Heterologous expression assays in yeast showed that MdSWEET23 functions in sucrose transport. The overexpression of *MdSWEET23* in the ‘‘Orin” calli increased the soluble sugar content. The silencing of *MdSWEET23* significantly reduced the contents of sucrose and sorbitol in apple fruits. Ectopic overexpression of *MdSWEET23* in tomato altered sugar metabolism and distribution in leaves and fruits, causing a reduction in photosynthetic rates and plant height, enhanced cold stress tolerance, and increased the content of sucrose, fructose, and glucose in breaking color fruits, but did not increase sugar sink potency of tomato fruits.

## Introduction

In higher plants, the synthesis, transport, and distribution of photosynthetic products are important physiological processes. For economically valuable fruits, phloem unloading and post-phloem transport play important roles in fruit quality improvement and yield formation (Patrick, 1997; Zhang et al., 2004; Ma et al., 2019). Zhang et al. (2018) pointed out that the transport of carbohydrates is more important than their synthesis during sugar accumulation in fruits. Therefore, enhancing the unloading capacity of sink organs is more beneficial for improving crop yield and quality than increasing the photosynthetic efficiency of source organs. For fruits that accumulate high levels of sugar, especially in later fruit development, the apoplasmic pathway facilitates continuous sugar accumulation and prevents sugar reflux from fruits (Zhang et al., 2004). For example, in apple (Zhang et al., 2004) and pear (Zhang et al., 2014), phloem unloading is the apoplasmic pathway throughout fruit development. Although phloem unloading is the symplasmic pathway in early fruit development in tomato (Ruan and Patrick, 1995) and grapes (Zhang et al., 2006), the apoplasmic unloading pathway is utilized in late fruit development. In Jujube fruits, a transient symplasmic unloading pathway exists in the middle of development, and the pathway in the early and late developmental stages is the apoplasmic pathway (Nie et al., 2010).

Sugar transporters have vital roles in the transmembrane transport of sugars. There are three types of sugar transporters in plants: monosaccharide transporters (MSTs), sucrose transporters (SUTs), and Sugars Will Eventually be Exported Transporters (SWEETs). Unlike the MSTs and SUTs, SWEET proteins are considered to be bidirectional uniporters by which sugar can be transported across the cell membrane along a concentration gradient without depending on pH and adenosine triphosphate (Chen, 2014; Chen H. Y. et al., 2015). SWEETs are involved in numerous plant biological processes, including growth, nectar secretion, seed nutrient filling, fruit development, and response to biotic and abiotic stresses (Chen, 2014; Yang et al., 2018; Wang S. et al., 2019; Zhang W. et al., 2019; Breia et al., 2021; Li et al., 2022). SWEETs mediate the transport of sugar from SE/CC complexes to sink tissues in apoplasmic phloem unloading. In Arabidopsis, AtSWEET9 has a distinct role in nectar secretion (Breia et al., 2021); AtSWEET11, AtSWEET12, and AtSWEET15 participate in seed development by mediating sucrose transport from the seed coat to the embryo (Breia et al., 2021). In tomato, glucose transporter *SlSWEET1a* is highly expressed in the veins of young leaves (sink) and plays a key role in glucose transport from the apoplast to the parenchyma cells (Ho et al., 2019). In cucumber, a hexose transporter CsSWEET7a that was localized to the phloem region of the flowers participates in the apoplasmic phloem unloading during flower anthesis. CsSWEET7a was also involved in sugar phloem unloading in cucumber fruit (Li et al., 2022). Hu et al. (2022) reported that CsSWEET2 plays an important role in improving plant cold tolerance. In tea plant, the expression levels of *CsSWEET16*, *CsSWEET1a*, and *CsSWEET17* are altered under cold stress (Wang et al., 2018; Yao et al., 2020). In rice, OsSWEET13 and OsSWEET15 are involved in the response to drought and salt stress by interacting with the ABA-responsive transcription factor OsbZIP72 (Mathan et al., 2021).

In our previous study, 27 *SWEETs* were identified from the apple genome, and MdSWEET1, 6, 8, 9, 10, 18, 20, 23, 26, and 27 may play roles at different fruit developmental stages (Nie et al., 2022). Zhen et al. (2018) reported that MdSWEET9b and MdSWEET15a may be involved in regulating sugar accumulation in apple fruits. MdSWEET9b, a sucrose transporter, influences fruit sugar accumulation by binding their promoters to MdWRKY9, which interacted with MdbZIP23/46 at the protein and DNA levels (Zhang et al., 2023). Our previous findings suggested that *MdSWEET23* is involved in sugar unloading in apple fruit, as the expression of it was positively correlated with the fruit sucrose, glucose, fructose, and soluble sugar contents. (Nie et al., 2022). In this study, we characterized the *MdSWEET23* gene in apple and found that ectopic overexpression of *MdSWEET23* in tomato improved the cold resistance in the seedling stage and increased sugar content in breaking color (BC) fruits.

## Materials and methods

### Plant materials and growth conditions

The apple fruit samples utilized in this study were collected from 10-year-old ‘‘Hanfu’’ apple trees (*M. domestica*) cultivated at the Shenyang Agricultural University experimental station (123.88°E, 41.88°N). The ‘Orin’ calli in this study were grown on MS medium with 0.8 mg·L^−1^ 6-BA and 1.5 mg·L^−1^ 2,4-D, in darkness at 25°C. *Nicotiana benthamiana* plants used for subcellular localization were cultivated with a 16 h/8 h (light/dark) photoperiod at 25 ± 2°C and a relative humidity of 50%–70%. In this study, tomato (*Solanum lycopersicum* L. ‘Micro-Tom’) seeds were used for genetic transformation. Both wild-type (WT) plants and transgenic tomato lines were grown in an artificial climate chamber under a 16 h/8 h (day/night) photoperiod with a temperature of 25 ± 2°C/18 ± 2°C (day/night) and a relative humidity of 50%–70%. All collected samples were immediately frozen in liquid nitrogen and stored at -80°C until use.

### RNA extraction, cDNA synthesis, and quantitative real-time PCR

RNA extraction, cDNA synthesis, and qRT-PCR were performed using our previously described method (Nie et al., 2022). All primers used are presented in **Table S1**. Gene expression levels were analyzed using the 2^-ΔΔCT^ method (Livak and Schmittgen, 2001). The experiment was repeated three times.

### Cloning of *MdSWEET23*, sequence alignment, and phylogenetic analysis

The sequence of *MdSWEET23* was acquired from the Genome Database for Rosaceae. The cDNA of *MdSWEET23* was amplified from ‘‘Hanfu’’ apple fruits. The pRI101-*MdSWEET23*-GFP recombinant plasmid was constructed for subcellular localization and genetic transformation. The sequence of *MdSWEET23* was submitted to the online websites MOTIF search (https://www.genome.jp/tools/motif/), DeepTMHMM (https://dtu.biolib.com/DeepTMHMM), and ProtParam tool (http://web.expasy.org/protaram/) for analysis of conserved motifs, transmembrane domains, molecular weight, theoretical isoelectric point (PI), and grand average of hydropathicity. Separate multiple sequence alignments were performed for MdSWEET23 and 17 *Arabidopsis* SWEET sequences (AtSWEET1-17) using ClustalW in MEGA X software. Subsequently, a phylogenetic tree was constructed using the Neighbor-joining method (Kumar et al., 2018).

### Subcellular localization of *MdSWEET23*

The pRI101-*MdSWEET23*-GFP fusion protein was transiently expressed in *Nicotiana benthamiana* and onion epidermal cells as previously described (Zhang et al., 2021). Empty vectors expressing non-targeted GFP were used as controls. Fluorescent signals were observed and photographed using a fluorescent microscope system (NikonNi-E, Nikon, Japan).

### Promoter cloning, sequence analysis, and GUS histochemical staining

An 1863 bp sequence upstream of the start codon (ATG) of the *MdSWEET23* gene was amplified from the apple genome DNA. The primers used for this extraction are listed in **Table S1**. The sequences of the cloned promoter were submitted to the PlantCARE website for online analysis of *cis*-acting elements. The PCR product was cloned into the pBI121 vector upstream of the β-glucuronidase (GUS) gene to construct the pBI121-*p*MdSWEET23**-GUS recombinant plasmid. The cDNA of *MdSWEET23* was cloned into the plasmid pBI121-p*MdSWEET23*-GUS downstream *p*MdSWEET23** to construct the pBI121-*p*MdSWEET23**-*MdSWEET23*-GUS plasmid. The recombinant plasmids were transformed into ‘‘Hanfu’’ apple fruits using the *Agrobacterium tumefaciens* LBA4404 and the method described by Spolaore et al. (2001). After 3 days of storage in the dark, the injected fruits were sampled for GUS histochemical staining. Subsequently, the tissues expressing GUS were examined and photographed.

### Yeast complementation assay

Yeast functional complementation assays were conducted following the methods described in a previous study (Loqué et al., 2007). Briefly, the recombinant plasmid pDR196-*MdSWEET23* was constructed, with the primers used in the construction process presented in **Table S1**. Subsequently, the recombinant plasmid pDR196-*MdSWEET23* or the empty pDR196 vector (as a control) was individually introduced into the yeast mutant strains, SUSY7/ura and EBY.VW4000. SUSY7/ura and EBY.VW4000 yeast cells carrying pDR196-*MdSWEET23* were inoculated on a solid SD/Ura medium supplemented with different carbon sources. Transformants were incubated at 30°C for 2–4 days and then observed and imaged.

### VIGS test

A 210 bp fragment of *MdSWEET23* cDNA fragment corresponding to bases 675 bp–885 bp of *MdSWEET23* was PCR amplified from ‘‘Hanfu’’ apple fruit cDNA. The PCR products obtained were successfully ligated into the pTRV2 vector. *Agrobacterium* strain GV3101 was separately transformed with pTRV1, pTRV2, and pTRV2-*MdSWEET23*. Subsequently, TRV1- and TRV2-**MdSWEET23* Agrobacterium* strains were co-inoculated into 140 days after bloom (DAB) ‘‘Hanfu’’ apple fruit by infiltrated using a needleless 1 mL syringe at the maximum diameter of apple fruit, with each fruit being injected three times at evenly distributed locations. The fruits injected with pTRV1 and pTRV2 *Agrobacterium* strains were used as controls. For each treatment, 10 fruits were injected and bagged. After 5 days, RNA was extracted from the samples for qRT-PCR analysis to detect the *MdSWEET23* silencing efficiency. Three fruits with high gene silencing efficiency were selected to determine sorbitol, fructose, glucose, and sucrose contents and *MdSDH5*, *MdSDH6*, and *MdSOT1* expression levels. The experiment was repeated three times.

### ‘‘Orin’’ calli transformation

The pRI101-*MdSWEET23*-GFP was transformed into ‘‘Orin’’ calli using the *Agrobacterium* strain LBA4404, and then the transformed calli was cultured on MS medium containing 50 mg·L^-1^ kanamycin for transgenic selection. The transgenic lines were confirmed using genomic PCR analysis with specific primers shown in **Table S1**. Transcript levels of *MdSWEET23* in these lines were examined using qRT-PCR. Samples were collected to determine the sorbitol, fructose, glucose, and sucrose contents. The experiment was repeated three times.

### Ectopic overexpression of *MdSWEET23* in tomato

**Figure 1:**
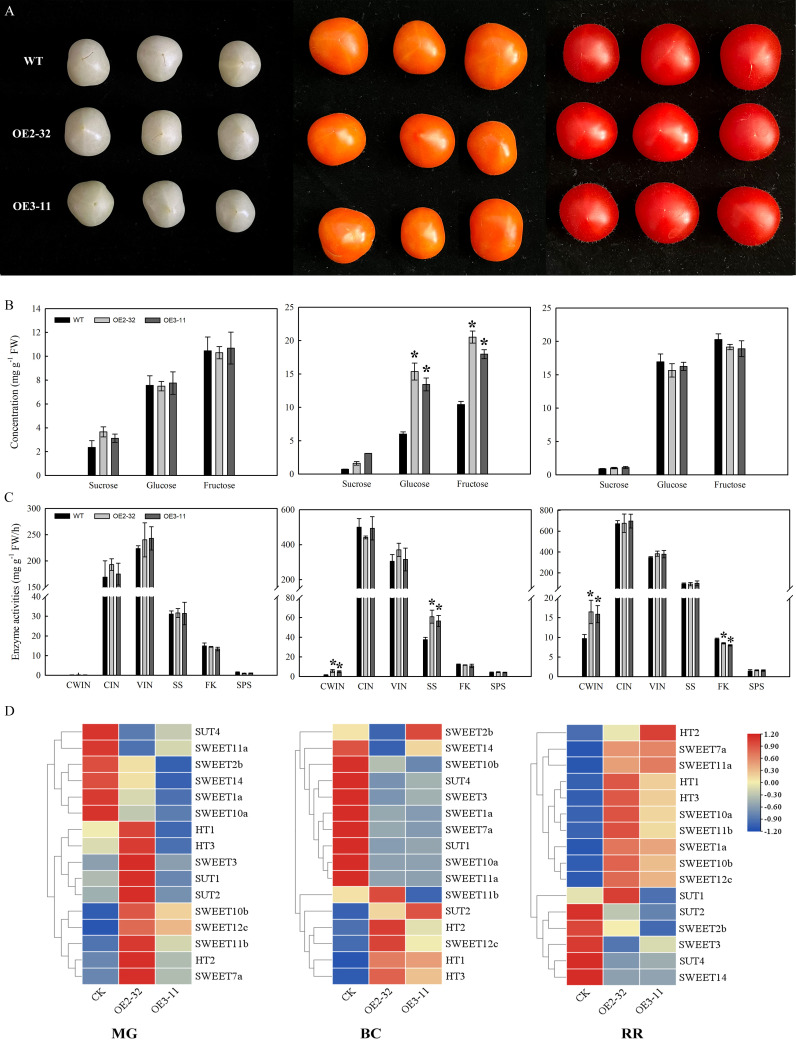
Sugar concentration, enzyme activities, and expression levels of sugar transporters in fruits of *MdSWEET23-*OE lines and WT. **(A)** The phenotype of MG, BC, and RR fruits of *MdSWEET23-*OE lines and WT. **(B)** Contents of sucrose, glucose, and fructose in MG, BC, and RR fruits of *MdSWEET23-*OE lines and WT. **(C)** Enzyme activities in MG, BC, and RR fruits of *MdSWEET23-*OE lines and WT. **(D)** Heat map illustrating the expression levels of sugar transporters in MG, BC, and RR fruits of *MdSWEET23-*OE lines and WT. The significance compared to WT was determined using Student’s t-test at *P<0.05.

Micro-tomato transformation was conducted using the methods described by Sun et al. (2006). Two transgenic lines of the T3 generation were selected and used for the phenotypic observation. Plant height was determined 60 days after seeding. Net photosynthetic rate (Pn) was determined using the portable photosynthesis system (CIRAS-2, PP systems, USA) at photosynthetically active radiation of 800 μmol·m^-2^·s^-1^. The leaf chlorophyll concentration was measured with a SPAD-502 meter (Konica Minolta Sensing, Japan) and expressed as SPAD values. Fruits at the mature green (MG), BC, and red ripe (RR) stages (**Figure 1A**) were used for analysis of sugar contents, analysis of sugar-metabolizing enzyme activities, and the expression levels of sugar transporter genes. The experiment was repeated three times.

### Cold treatments

Seedlings of the WT and two *MdSWEET23*-overexpressing (OE) transgenic plants were grown under normal conditions for 60 days, followed by cold treatment (3.5°C) for 3.5–4 h. Leaves were collected to measure the levels of relative electrolyte leakage (REL), soluble sugar, and starch. The REL was determined by the method of Hu et al. (2022). The experiment was repeated three times.

### Enzyme assays and determination of sugars and starch

The enzymatic activities of cell wall invertase (CWIN), sucrose synthase (SS), cytosolic invertase (CIN), fructokinase (FK), vacuolar invertase (VI), and sucrose phosphate synthase (SPS) were measured following the kit instructions of Suzhou Grace Biotechnology Co. The soluble sugar and starch content were determined by the methods of Zhang et al. (2021). The sorbitol, fructose, glucose, and sucrose contents were determined using high-performance liquid chromatography (HPLC) (1260 series, Agilent Technologies), a method referenced by Li et al. (2020).

### Statistical analysis

All experiments were conducted with three independent biological replicates. Error bars represent the standard deviation (SD) of the three replicates, and values are presented as the mean ± SD. All statistical analyses were performed using IBM SPSS Statistics 23. Additionally, a t-test for independent samples was used for significant differences between the two groups of data, and significant differences were expressed as * P<0.05. One-way analysis of variance was performed on the data using Duncan`s multiple range test, and the results are indicated by letters. Histograms were plotted using Sigma Plot 10.0.

## Results

### *MdSWEET23* encodes a Clade III SWEET protein

**Figure 2:**
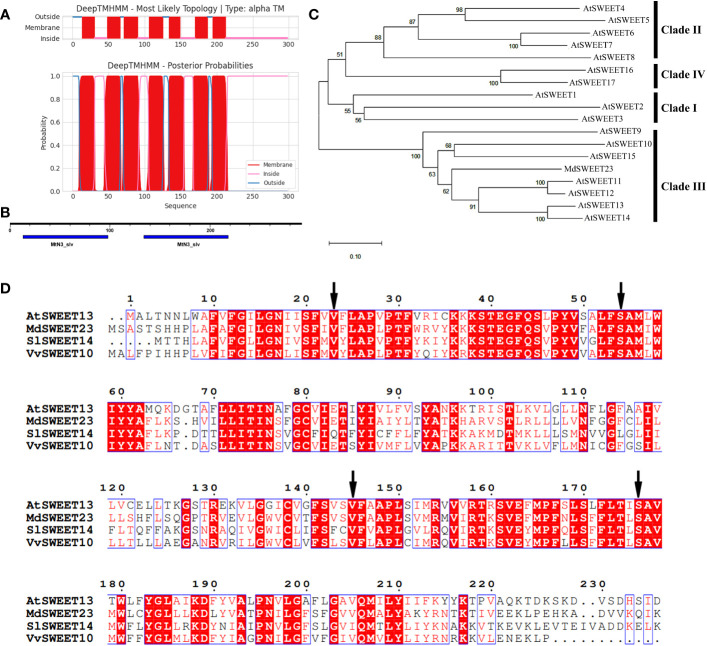
Sequence analysis of *MdSWEET23*. **(A)** TMs of the *MdSWEET23* protein. **(B)** Conserved motifs of *MdSWEET23*. **(C)** Phylogenetic analysis of SWEET proteins between *MdSWEET23* and AtSWEETs (AtSWEET1-17). **(D)** Multiple sequences alignment of SWEET proteins from *Malus domestica* (*MdSWEET23*), *Arabidopsis thaliana* (AtSWEET13), *Solanum lycopersicum* (SlSWEET14), and *Vitis vinifera* (VvSWEET10). The arrow indicates the position of Leu, Asn, Met, and Asn.

The *MdSWEET23* cDNA was cloned from the total RNA of “Hanfu” apple fruits, and it consists of an 897 bp open reading frame that encodes a polypeptide of 298 amino acids (**Table S2**). The polypeptide has a molecular weight, an isoelectric point, and a grand average hydropathicity of 33.48 kDa, 8.82, and 0.497, respectively (**Table S3**). MOTIF search and TMHMM analysis showed that MdSWEET23 contained seven α-helical transmembrane domains with two conserved MtN3/saliva motifs (**Figures 2A, B**), which are unique to the typical plant SWEET proteins. Phylogenetic analysis revealed that MdSWEET23 has the highest similarity to AtSWEET13 with a similarity of approximately 50.50% (**Table S4**), and MdSWEET23 is classified as a member of the Clade III in the SWEET family (**Figure 2C**). Han et al. (2017) reported the 2.8-Å resolution crystal structure of AtSWEET13 in the inward-facing conformation with a substrate analog, 2′-deoxycytidine 5′-monophosphate, bound in the central cavity. Four residues (Val23TM1, Ser54TM2, Val145TM5, and Ser176TM6) inside the binding pocket in AtSWEET13 lead to a larger cavity than monosaccharide transporters, which have Leu, Asn, Met, and Asn in the corresponding sites, and these residues are most likely involved in substrate selectivity. MdSWEET23 has the same residues as AtSWEET13 in the corresponding sites by amino acid sequence alignment (**Figure 2D**). Thus, it is assumed that the transport activity of MdSWEET23 toward sucrose may be higher than that toward glucose.

### Identification of promoter and cis-regulatory elements of *MdSWEET23*

The promoter regions (1863 bp) of *MdSWEET23* were cloned (**Table S5**), and its *cis*-acting elements were analyzed using the PlantCARE program. Hormone-, stress-, and light-responsive and organ-specific *cis*-acting elements were identified in the *MdSWEET23* promoter. Thus, it is speculated that the expression of *MdSWEET23* may be in response to light, hormones (such as MeJA, auxin, and ABA), and abiotic stresses (such as low-temperature and drought). Phloem tissue-specific *cis*-acting elements (as-1) were also identified in the *MdSWEET23* promoter, which implied that it might be a phloem-specific promoter (**Table 1**).

**Table 1:** *Cis-*elements were predicted in the promoter regions of *MdSWEET23*.

Site Name	Sequence	No.	Function
TGA-element	AACGAC	1	auxin responsiveness element
MYC	CATTTG	3	drought responsiveness element
as- 1	TGACG	4	phloem tissue-specific element
CGTCA-motif	CGTCA	4	the MeJA-responsiveness element
TGACG-motif	TGACG	4	the MeJA-responsiveness element
ABRE	ACGTG	5	the abscisic acid responsiveness element
DRE core	GCCGAC	1	a dehydration-responsive element
MBS	CAACTG	1	drought responsiveness element
WRE3	CCACCT	1	wound responsive elements
ARE	AAACCA	1	the anaerobic induction element
LTR	CCGAAA	1	low-temperature responsiveness element
CAT-box	GCCACT	2	meristem expression regulatory element

### Spatial expression of *MdSWEET23*

**Figure 3:**
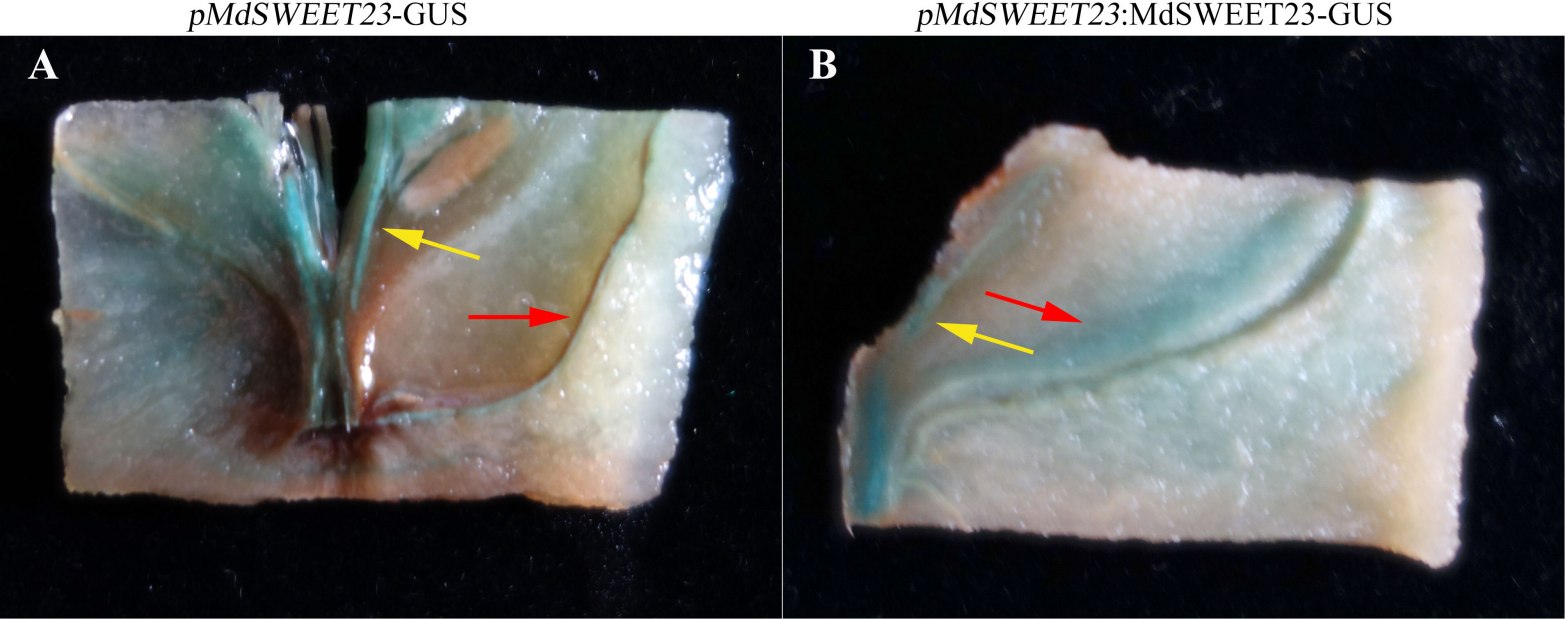
Spatial expression of *MdSWEET23*. **(A)** *pMdSWEET23*-GUS staining pattern in ‘‘Hanfu’’ apple fruit. **(B)** *pMdSWEET23*:*MdSWEET23*-GUS staining pattern in ‘‘Hanfu’’ apple fruit. The red arrows indicate the sepal vascular bundle and the yellow arrows indicate the carpel vascular bundle.

To confirm the spatial expression patterns of *MdSWEET23*, we fused its promoter sequence with the GUS gene to generate two constructs, *p*MdSWEET23**-GUS and *p*MdSWEET23**:*MdSWEET23*-GUS. The GUS activity in transgenic apple fruits was predominantly detected within the vascular bundle region, particularly in the sepal and carpel vascular bundles (**Figure 3**).

### MdSWEET23 is localized on the plasma membrane

**Figure 4:**
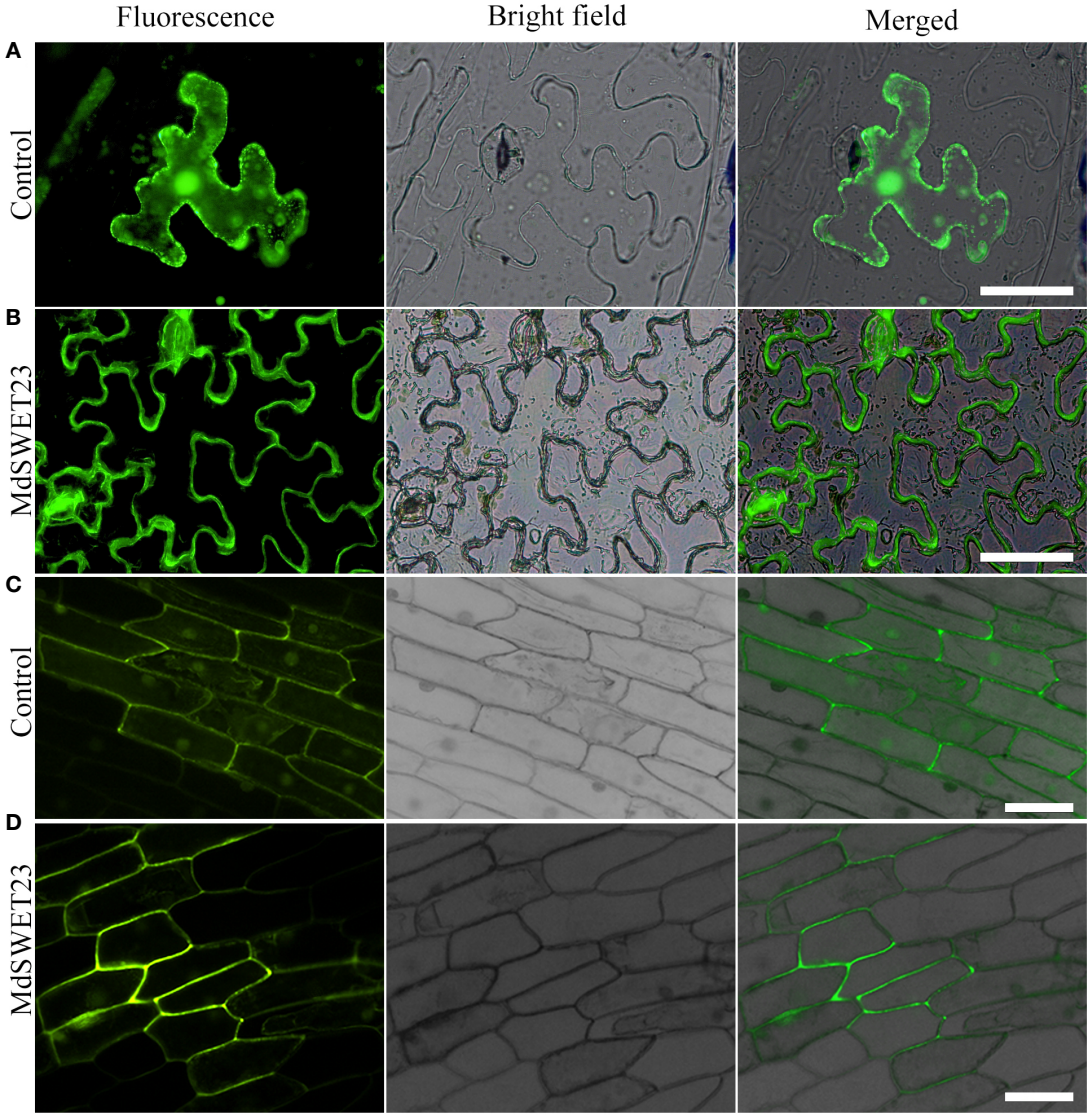
Subcellular localization of *MdSWEET23*. **(A, B)** *MdSWEET23*-GFP fusion protein was transiently expressed in tobacco leaves. **(C, D)** *MdSWEET23*-GFP fusion protein was transiently expressed in onion epidermal cells. Transient expression of GFP alone (35S: GFP) was used as a control. The scale bar in the figure is 100 μm.

To determine the subcellular localization of MdSWEET23, a *MdSWEET23*-GFP fusion protein was transiently expressed in both *Nicotiana benthamiana* and onion epidermal cells. Fluorescence signals from the *MdSWEET23*-GFP fusion protein were only detected on the plasma membrane in *Nicotiana benthamiana* and onion epidermal cells (**Figure 4B, D**), indicating that MdSWEET23 is likely localized on the plasma membrane.

### *MdSWEET23* transports sucrose in yeast

**Figure 5:**
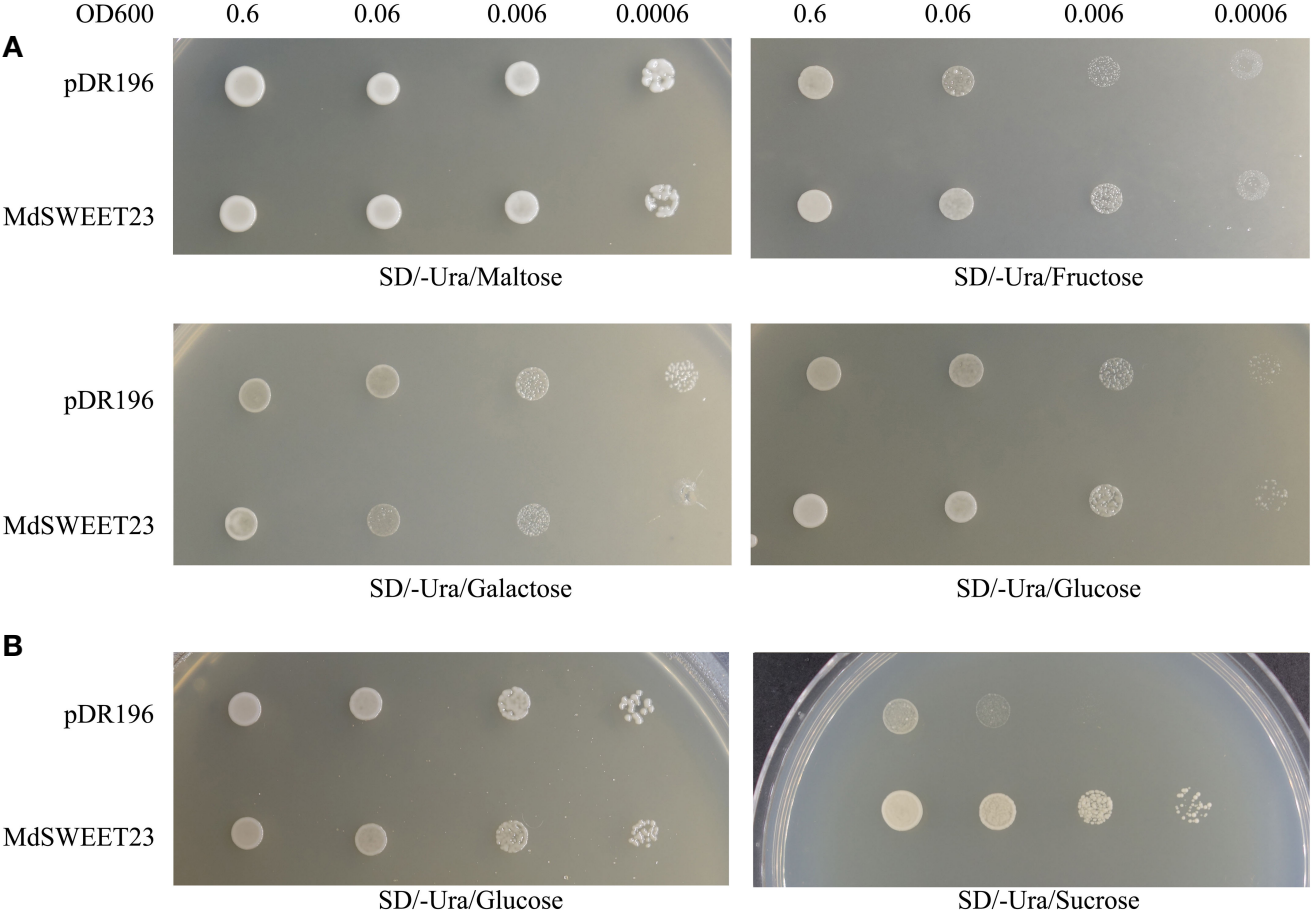
Heterologous expression of *MdSWEET23* in yeast strain EBY.VW4000 and SUSY7/ura. **(A)** Yeast cells EBY.VW4000 with pDR196-MdSWEET23 or pDR196 vector (as a negative control) were grown on (SD)/-uracil (Ura) solid medium containing 2% (V/W)maltose, 2% (V/W)fructose, 2% (V/W)galactose, or 2% (V/W)glucose as sole carbon source. **(B)** Yeast cells SUSY7/ura with pDR196-MdSWEET23 or pDR196 vector (as a negative control) were grown on SD/-Ura solid medium containing 2% (V/W) glucose, or 2% (V/W) sucrose as sole carbon source.

The functionality of MdSWEET23 in sugar transport was examined by transforming yeast strains, including EBY.VW4000 and SUSY7/ura3 with the pDR196-*MdSWEET23* fusion vector. The yeast mutant strain, EBY.VW4000 displayed the inability to grow on monosaccharides but exhibited growth on maltose (Wieczorke et al., 1999). In contrast, the mutant strain SUSY7/ura3 could not use external sucrose as the sole carbon source (Riesmeier et al., 1992). A spotting assay showed that EBY.VW4000 cells transformed with pDR196-*MdSWEET23* and the empty pDR196 vector (as negative control) exhibited comparable growth rates on the culture medium containing 2% (w/v) maltose, galactose, glucose, and fructose (**Figure 5A**). The SUSY7/ura3 cells transformed either with pDR196-*MdSWEET23* or the empty vector pDR196 could grow well on 2% (w/v) glucose, whereas pDR196-*MdSWEET23* transformants grew better than the negative control when given 2% (w/v) sucrose (**Figure 5B**). These results indicate that MdSWEET23 is involved in sucrose transport.

### Silencing *MdSWEET23* influences the sugar accumulation in apple fruits

**Figure 6:**
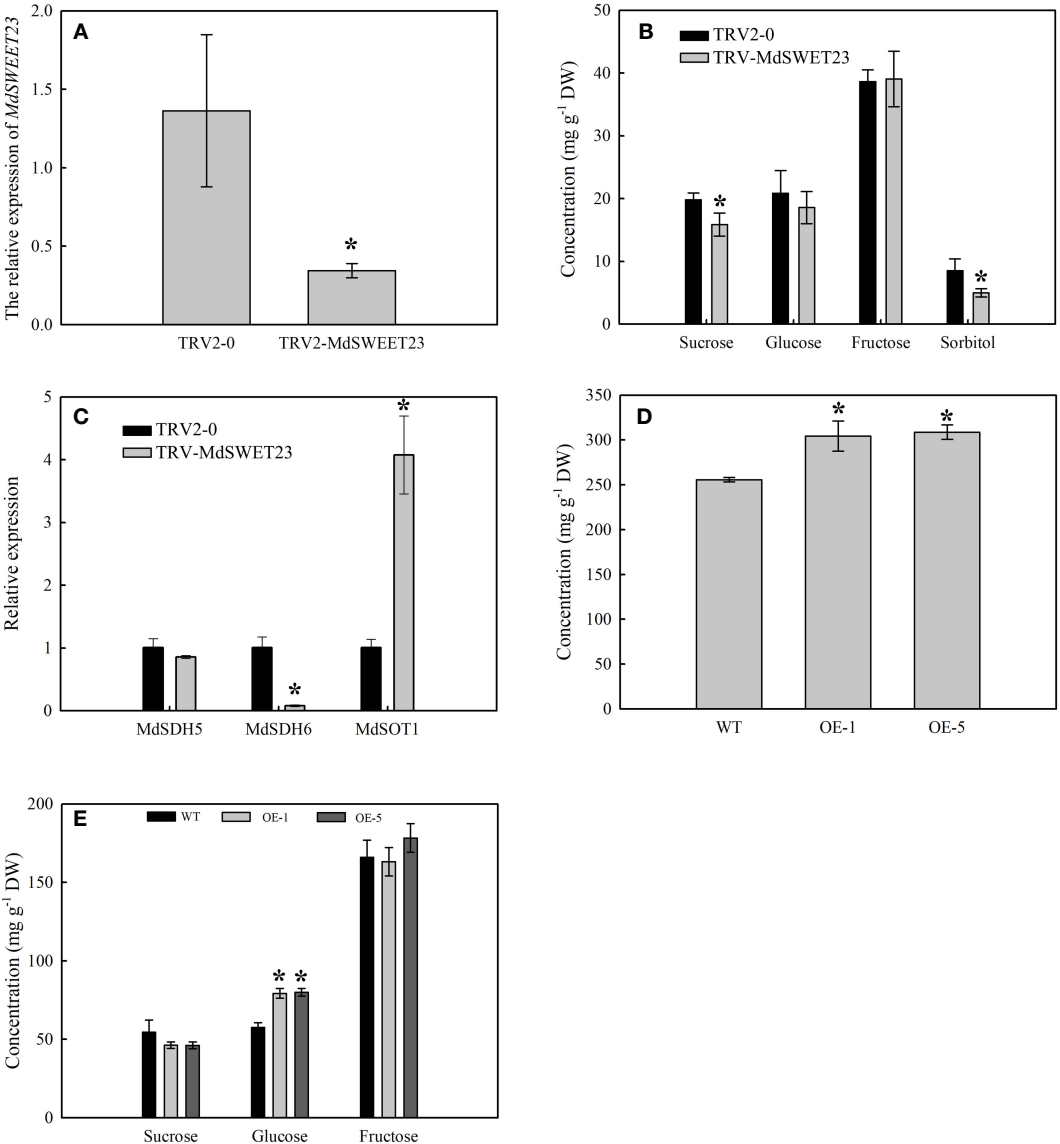
Functional analysis of MdSWEET23. **(A–C)** The expression levels of *MdSWEET23* **(A)**, *MdSDH5*, *MdSDH6*, and *MdSOT1* **(C)**, and the sugar content **(B)** in *MdSWEET23* silencing apple fruits. **(D, E)** The content of total soluble sugar **(D)** and the contents of sucrose, glucose, and fructose **(E)** in *MdSWEET23-*OE ‘‘Orin’’ calli. The significance compared to WT was determined using Student's t-test at *P<0.05.

The expression of *MdSWEET23* in VIGS-treated fruits was examined using qRT-PCR. The results showed that VIGS treatment resulted in a significant reduction in the transcript level of the *MdSWEET23*, which was only 25.28% of the control level (**Figure 6A**). Silencing of *MdSWEET23* significantly reduced sucrose and sorbitol contents in fruits; however, it had no significant effect on the accumulation of fructose and glucose (**Figure 6B**). *MdSWEET23* silencing resulted in a significant increase in the expression of *MdSOT1*, which was approximately 4 times higher than that of the control, while the expression of *MdSDH6* was significantly decreased (**Figure 6C**). MdSOT1 functions in the unloading of sorbitol in apple fruits (Wei et al., 2014). While, *MdSDH6* encodes a sorbitol dehydrogenase (SDH) that converts sorbitol to fructose (Wang et al., 2009). Therefore, the silencing of *MdSWEET23* not only reduced the accumulation of sucrose in ‘‘Hanfu’’ apple fruits but also affected the transport and metabolism of sorbitol.

### Overexpression of *MdSWEET23* increased the soluble sugar content in the ‘‘Orin” calli

A pRI101-*MdSWEET23* recombinant plasmid was constructed and transformed into ‘‘Orin” calli to verify the function of *MdSWEET23*. After the PCR identification, four overexpression lines were obtained, and OE-1 and OE-5 were selected for further experiments (**Supplementary Figure 1A**). The growth of ‘‘Orin” calli lines of OE-1 and OE-5 is shown in **Supplementary Figure 1B**. The contents of soluble sugars and glucose in both OE-1 and OE-5 lines were significantly higher than those in the WT plants (**Figures 6D, E**). Since only sucrose was added as a carbon source in the medium, the accumulation of glucose in the calli might be due to sucrose hydrolysis. The overexpression of *MdSWEET23* in the “Orin” calli promoted the accumulation of soluble sugars.

### *MdSWEET23* overexpression reduced plant height and photosynthetic rates in tomato

**Figure 7:**
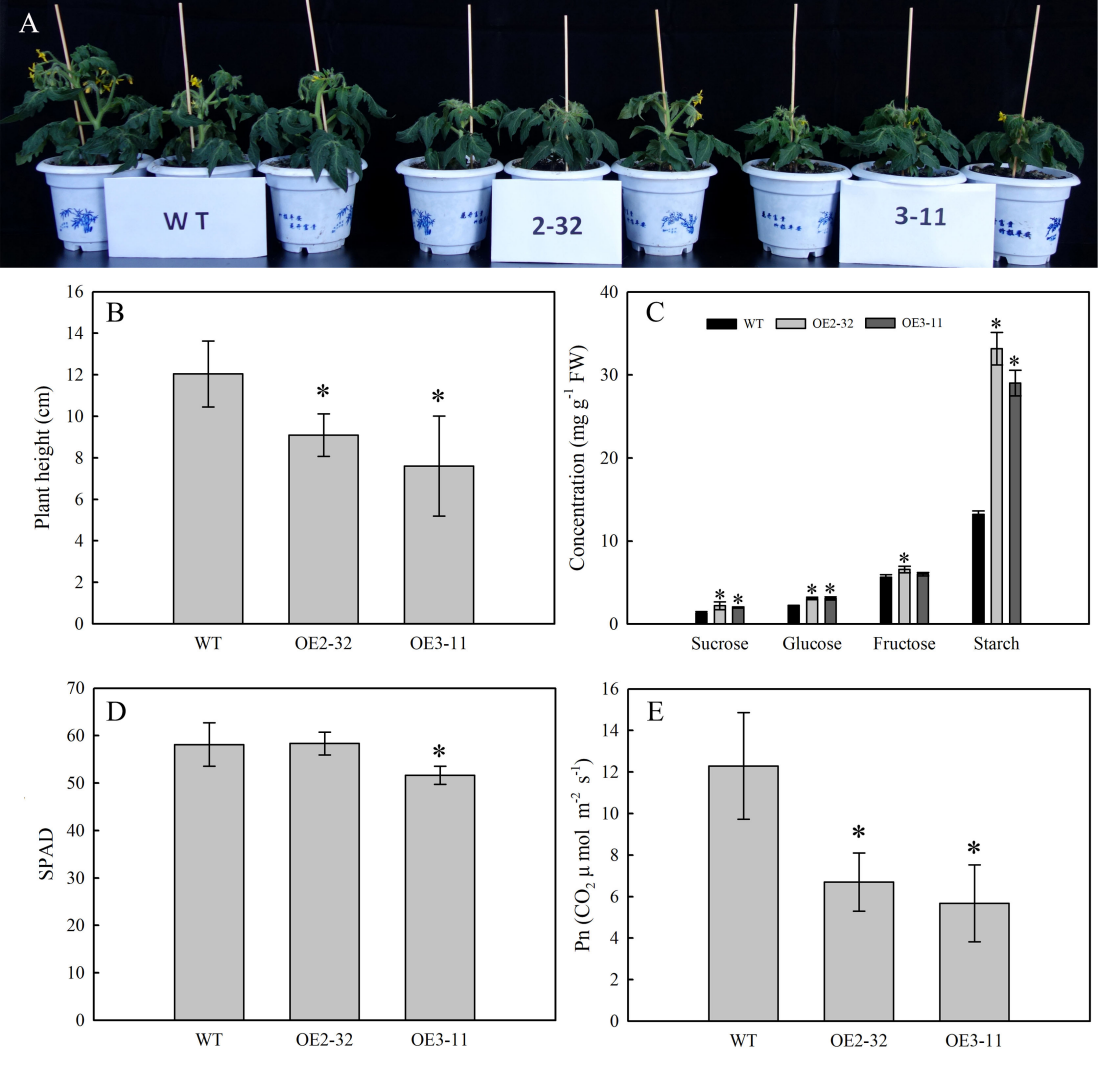
Phenotype and physiological characterization of *MdSWEET23*-OE tomato lines. **(A)** Phenotypic characteristics of *MdSWEET23*-OE lines and WT. **(B)** Plant height of *MdSWEET23*-OE lines and WT. **(C)** Contents of sugar and starch in leaves of *MdSWEET23*-OE lines and WT. **(D)** Leaf SPAD values of *MdSWEET23*-OE lines and WT. **(E)** Pn values of *MdSWEET23*-OE lines and WT. The significance compared to WT was determined using Student's t-test at *P<0.05.

To determine the impact of *MdSWEET23* expression on sugar accumulation in plants, transgenic tomato lines overexpressing *MdSWEET23* driven by the CaMV35S promoter were generated. After PCR identification, eight *MdSWEET23*-overexpression (*MdSWEET23*-OE) lines were obtained (**Supplementary Figure 2**), and OE2-32 and OE3-11 in T3 generation were used for further experiments. Compared with WT plants, the plant height in lines OE2-32 and OE3-11 decreased by 24.42% and 36.88%, respectively (**Figures 7A, B**), and Pn of mature leaves decreased by 45.43% and 53.84%, respectively (**Figure 7E**). The contents of sucrose, glucose, fructose, and starch in mature leaves of lines OE2-32 and OE3-11 were significantly higher than those in WT plants. Particularly, the starch concentration in these lines was 2.51 and 2.19 times higher than that in WT plants, respectively (**Figure 7C**).

### *MdSWEET23* overexpression in tomato results in sugar accumulation increased in BC fruits

We examined the sugar content in MG, BC, and RR fruits (**Figure 1A**) of *MdSWEET23*-OE lines and WT. The sucrose content in MG fruits of OE2-32 and OE3-11 was significantly higher than that of WT, but no significant differences were observed in glucose and fructose contents between them (**Figure 1B**). At the BC stage, *MdSWEET23*-OE lines exhibited significantly higher levels of sucrose, glucose, and fructose than the WT (**Figure 1B**). In contrast, the sucrose, glucose, and fructose contents of RR fruits showed no significant difference between *MdSWEET23*-OE lines and WT (**Figure 1B**).

To investigate the reasons for the sugar level alteration in *MdSWEET23-*OE lines, we further examined the activity of sugar metabolism-related enzymes and the expression levels of genes related to sugar transport. In MG fruits, no significant difference was found in enzyme activities between *MdSWEET23*-OE lines and WT (**Figure 1C**). In BC fruits, the CWIN and SS activities of *MdSWEET23*-OE lines were significantly higher than those of the WT, with 3.84 and 1.56 times increases, respectively. In RR fruits, the CWIN activity of *MdSWEET23*-OE lines was significantly higher than that of the WT, with a 1.66-fold increase, while the FK activity was significantly lower than that of the WT, at approximately 86% of the WT.

Compared with WT, the *HT1* and *HT2* expression levels in *MdSWEET23*-OE lines were notably higher in BC fruits, while no significant changes were observed in their expression in RR and MG fruits. In BC fruits of *MdSWEET23*-OE lines, the expression of *SUT2* was upregulated, while the expression of *SUT1* was downregulated; and the transcript levels of *SUT4* were notably downregulated in MG, BC, and RR fruits of the *MdSWEET23*-OE lines. The overexpression of *MdSWEET23* also affected the expression of several *SWEETs* (**Figure 1D**).

### *MdSWEET23* overexpression in tomato improves tolerance to cold stress at the seedling stage

**Figure 8:**
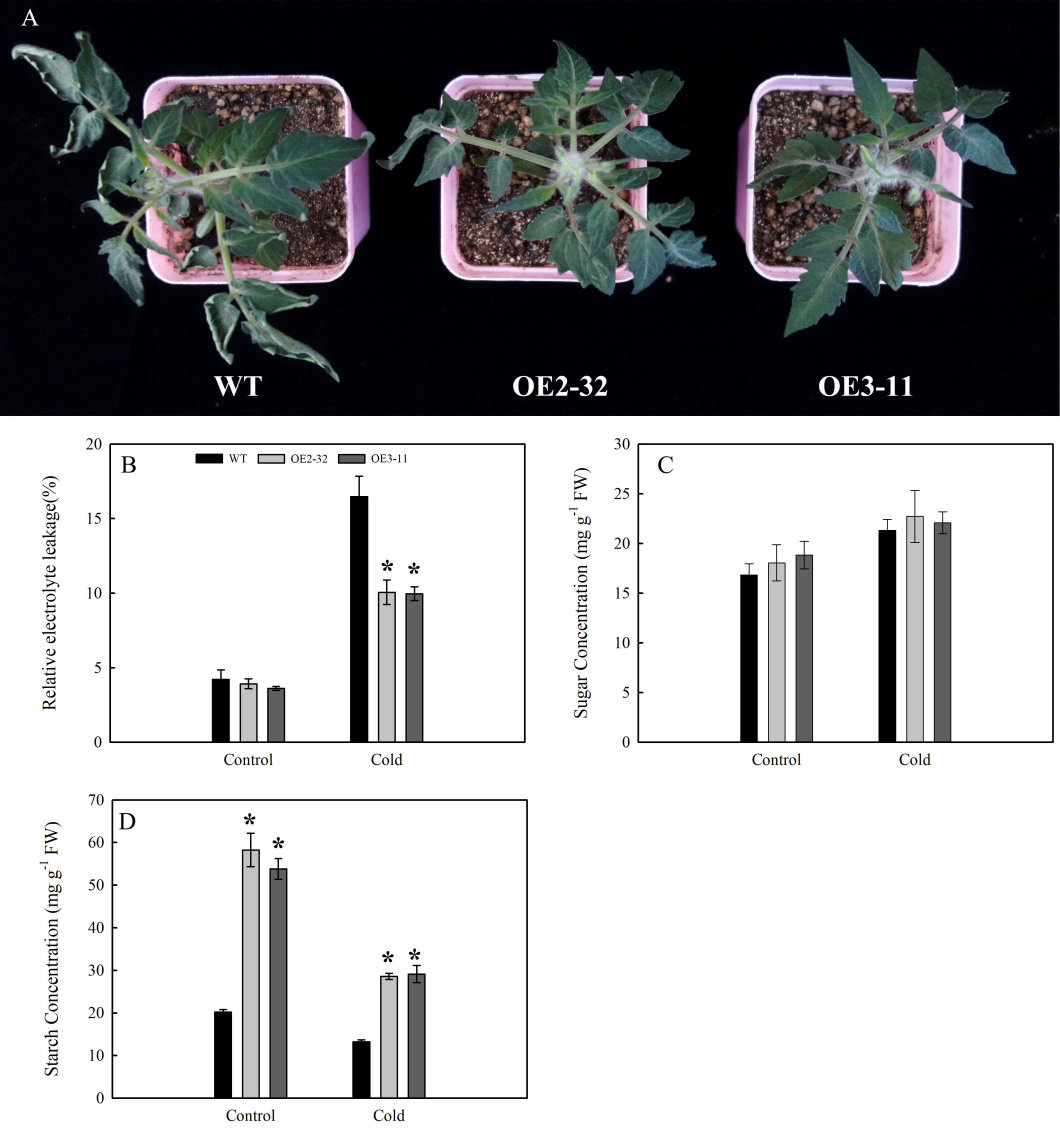
Changes in growth and physiological characteristics of *MdSWEET23*-OE lines and WT under cold treatment. **(A)** Phenotypic changes of *MdSWEET23*-OE lines and WT plants under cold treatment. **(B)** REL levels of *MdSWEET23*-OE lines and WT plants under cold treatment. **(C)** Content of total soluble sugars in leaves of *MdSWEET23*-OE lines and WT plants under cold treatment. **(D)** Content of starch in leaves of *MdSWEET23*-OE lines and WT plants under cold treatment. The significance compared to WT was determined using Student's t-test at *P<0.05.

Under cold stress (3.5°C for 4 h), no significant change was found in the leaves of OE2-32 and OE3-11 lines; however, the leaves of WT showed wilting symptoms (**Figure 8A**). The release of electrolytes can characterize the damage to plant cells (Hu et al., 2022). Before cold stress treatment, there was no significant difference in REL levels between the two transgenic lines and WT (**Figure 8B**). After cold treatment, the REL levels of OE2-32 and OE3-11 lines were lower than those of WT (**Figure 8B**). We also compared the changes in sugar and starch contents of leaves between *MdSWEET23*-OE lines and WT before and after cold treatment. The results showed that the soluble sugar content in leaves of *MdSWEET23*-OE lines was only slightly higher than that of WT under cold treatment (**Figure 8C**). The starch content in leaves of *MdSWEET23*-OE lines and WT all decreased when exposed to cold treatment; however, the starch content in leaves of OE2-32 and OE3-11 decreased from 58.27 to 28.61 mg·g^-1^ FW (by 50.89%) and from 53.81 to 29.16 mg·g^-1^ FW (by 45.81%), respectively, whereas that of WT only decreased from 20.27 to 13.23 mg·g^-1^ FW (by 34.73%) (**Figure 8D**).

## Discussion

### *MdSWEET23* is localized on the plasma membrane in vascular bundle tissues and involved in sucrose transport in apple

Fruit growth is a high-priority sink in assimilate partitioning. An apoplasmic phloem unloading strategy is employed in the fleshy fruits of apples, which requires sugar transporters to export sugar into the apoplasm. Many SWEETs function in flesh fruits, such as SlSWEET7a and SlSWEET14 in tomato (Zhang et al., 2021), VvSWEET10 in grape (Zhang Z. et al., 2019), and MdSWEET9b in apple (Zhang et al., 2023). CsSWEET7a localized on the plasma membrane in companion cells (CCs) of the phloem has recently been reported to be involved in sugar phloem unloading in cucumber fruit by exporting hexoses from CCs to the apoplasmic space (Li et al., 2021). In our previous study, *MdSWEET23* could play an important role during the progress of apple fruits maturation with starch breaks down and soluble sugars accumulate massively (Nie et al., 2022). Here, *MdSWEET23*, isolated from a cDNA library of apple fruits, belongs to clade III and is phylogenetically most closely related to AtSWEET13 (**Figure 2**). AtSWEET13, which also belongs to clade III, is plasma membrane-localized and transports both sucrose and GA (Kanno et al., 2016). But a recent study found that sucrose rather than GA transported by AtSWEET13 and AtSWEET14 directly affects anther development and seed yield (Wang et al., 2022). Our heterologous expression assay in yeast showed that MdSWEET23 functioned in sucrose transport rather than glucose and fructose transport and could restore SUSY7/ura3 growth on media supplemented with sucrose (**Figure 5**). Moreover, the overexpression of *MdSWEET23* in ‘‘Orin” calli promoted sugars accumulation in calli, which grown on MS medium with sucrose as the carbon source (**Figure 6**). These results indicate that MdSWEET23 is a functional sucrose transporter. In previous studies, it has been found that most SWEETs in clade III are capable of transporting sucrose. Such as AtSWEET11, 12, and 13 in *Arabidopsis* (Chen et al., 2012; Han et al., 2017), SbSWEET13a, 13b, and 13c in sorghum, and StSWEET11 in potato (Abelenda et al., 2019), but their functions are different.

The functions of SWEETs also largely depend on their subcellular and tissue localization. SWEET proteins are mainly localized on the plasma and the tonoplast, while, a few members are also localized on the endoplasmic reticulum and the Golgi membranes (Chen L. Q. et al., 2015; Jeena et al., 2019; Breia et al., 2021). AtSWEET13 (Kanno et al., 2016) in *Arabidopsis*, PbSWEET4 (Ni et al., 2020) and PuSWEET15 (Li et al., 2020) in pear, BsSWEET15 and BsSWEET16 (Lu et al., 2022) in *Bletilla striata*, and SlSWEET14 (Zhang et al., 2021) in tomato, are also localized on the plasma membrane. The localization of sugar transporter ClVST1 shifted from tonoplast in wild-type watermelon to plasma membrane in sweet watermelon, which increased sugar sink potency (Ren et al., 2020). MdSWEET23 was localized on the plasma membrane (**Figure 4**) and mainly expressed in the vascular bundle, especially in the sepal and carpel vascular bundles of apple fruit (**Figure 3**). The carpel vascular bundle is the major channel for nutrients transporting toward the seeds, and the sepal vascular bundle is the main channel for nutrient and water transport for fruit expansion (Wang et al., 2015). In *Arabidopsis*, *AtSWEET11, 12, and 15* are all highly expressed in the seed coat and play key roles in seed development (Le Hir et al., 2015). Therefore, it is speculated that MdSWEET23 may participate in apple seed development. The expression of MdSWEET23 in the sepal vascular bundle region indicates that it may be involved in the unloading of sucrose to the fleshy tissue of apple fruits. In apple, MdSWEET9b functions on the SE/CC, and its surrounding parenchyma cells is involved in fruit sugar accumulation (Zhang et al., 2023). In cucumber, CsSWEET7a, localized to the plasma membrane in CCs of the phloem, is involved in sugar phloem unloading in cucumber fruit by removing hexoses from CCs to the apoplasmic space (Li et al., 2021).

The silencing of *MdSWEET23* in “Hanfu” apple fruits significantly reduced sucrose and sorbitol content, which further confirmed the involvement of *MdSWEET23* in sucrose transport and accumulation in apple fruits. Further, whether the decrease in sorbitol content (**Figure 6B**) was due to *MdSWEET23* silencing and whether MdSWEET23 was involved in sorbitol transport and accumulation in apple fruits? The expression of *MdSOT1* significantly increased, whereas that of *MdSDH6* substantially decreased in the *MdSWEET23*-silenced fruits. These results indicate that *MdSWEET23* silencing affects sorbitol metabolism and transport in apple fruits. However, more direct evidence is needed to prove whether MdSWEET23 can transport sorbitol. In apple, the carbohydrates used for long-distance transportation are sucrose and sorbitol. Sucrose and sorbitol arriving in the SE/CC complex are pumped into the apoplasmic space by SUT/SOT (Fan et al., 2009; Chen et al., 2012) after that sucrose is broken down to hexose by CWIN, which was transported to the phloem parenchyma cells by the hexose transporters (Zhang et al., 2004). We speculate that MdSWEET23 may play a role in sucrose or sorbitol transport along the sugar concentration gradient from the apoplasmic space to the phloem parenchyma cells.

### Ectopic overexpression of *MdSWEET23* in tomatoes caused altered sugar metabolism and distribution

Sugar transport can influence the distribution of carbohydrates throughout the plant, which is important for plant growth, and the disorganization of sugar distribution will lead to abnormal plant growth. The overexpression of *ZjSWEET2.2* in jujube led to a significant increase in leaf photosynthetic assimilate (Geng et al., 2020), whereas the overexpression of *PbSWEET4* in strawberry reduced sugar content in leaves (Ni et al., 2020). Klemens et al. (2013) found that the overexpression of *AtSWEET16* in *Arabidopsis* resulted in significantly increased growth compared to the WT. While, overexpression of *OsSWEET14* and *OsSWEET5* in rice significantly reduced plant height (Zhou et al., 2014; Kim et al., 2021). Silencing of *SlSWEET7a* and *SlSWEET14* in tomato resulted in a 35%–48% increase in plant height compared to the WT (Zhang et al., 2021). The ectopic overexpression of *MdSWEET23* in tomato led to an over-accumulation of carbohydrates in leaves, with a decrease in photosynthetic rate and plant height (**Figure 7**). In apple, *MdSWEET23* is expressed mainly in the sepal and carpel vascular bundles of fruits (sink organs) (**Figure 3**). While, in transgenic tomato lines, the expression of the *MdSWEET23* was driven by the CaMV35S promoter, a constitutive promoter, and thus *MdSWEET23* expression in tomato lacks temporal and spatial regulation. In source organs, SWEETs are mainly responsible for transporting sucrose from the synthesis site to the loading site (Chen et al., 2012). The expression of *MdSWEET23* in tomato functional leaves (source organ) may have affected the loading process of photosynthetic assimilates, too much sucrose was transported to the loading site and caused the abnormal accumulation of starch and soluble sugars in mature leaves. The increases in soluble sugar and starch content in leaves negatively regulate the photosynthetic rate, causing a decrease in the net photosynthetic rate of the leaves (Sonnewald, 2001). In maize, CST1 belongs to the Clade I of the SWEET protein family, is specifically expressed in subsidiary cells, and positively regulates stomatal opening and source capacity at the grain-filling stage (Wang H. et al., 2019). At the seedling stage, phenotypes of the *MdSWEET23*-OE plants might partially or wholly be ascribed to the disordered sugar distribution.

Previous genome-wide studies in different plants have found that *SWEETs* exhibit variable expression patterns under cold treatments, which suggests that SWEETs are involved in cold-induced sugar-signaling responses (Zhang W. et al., 2019; Jiang et al., 2021). Overexpression of *AtSWEET16* (Klemens et al., 2013) and *AtSWEET17* (Chardon et al., 2013) in *Arabidopsis* elevated sugar content and increased cold tolerance. Hu et al. (2022) found that cold-tolerant cucumber had a higher expression level of *CsSWEET2* than cold-sensitive cucumber, and the overexpression of *CsSWEET2* in *Arabidopsis* enhanced the cold resistance. The *MdSWEET23*-OE lines showed higher resistance to cold stress. Ectopic overexpression of *MdSWEET23* accelerated starch degradation in leaves under cold stress, but leaf soluble sugar content in OE lines showed no significant difference with WT. We speculated that the overexpression of *MdSWEET23* in tomato may have resulted in facilitating the process of sugar loading during cold treatment, which in turn allowed more sugar to be transported out of the source organ, and could potentially influence starch hydrolysis through the feedback regulation mechanism. Overexpression of *MdSWEET23* in tomato may alter sucrose transport and distribution in OE lines under cold stress, thereby maintaining sugar homeostasis in response to cold stress. The possible mechanisms of increased cold tolerance of *MdSWEET23*-OE tomato lines need to be further investigated.

Based on the characteristics of *MdSWEET23* in substrate transport, subcellular and tissue localization (**Figures 3**–**5**), we speculated that *MdSWEET23* could be involved in the uptake of sucrose through the plasma membrane in sepal and carpel vascular bundles of apple fruits. Ectopic overexpression of *MdSWEET23* in tomato significantly increased the sugar content of the BC fruits but had no significant effect on the sugar content of the RR fruits (**Figure 1**). In pear, overexpression of *PuSWEET15* led to a significant increase in sucrose content in fruits (Li et al., 2020). Similarly, overexpression of *VvSWEET10* resulted in a significant enhancement of sugar content in grape calli and tomato fruits (Zhang Z. et al., 2019). Silencing of *SlSWEET7a* and *SlSWEET14* in tomato increased the sugar content of the fruits(Zhang et al., 2021). Sugars can act as signaling molecules that control distinct aspects of plant development. Several recent studies have reported that downstream sugar transporters could affect the sugar metabolism, by influencing upstream sugar metabolic enzyme activities and expression of sugar transporters through the feedback regulation mechanisms. For example, in cucumber, the overexpression of *CsSWEET7a* could shift the enzymatic reactions to produce more hexoses due to its overexpression leading to more hexose being unloaded from CCs, and less hexose retained in the cytosol of CCs (Li et al., 2021). MdERDL6-1, a tonoplast H+/glucose symporter, was highly expressed in apple fruits, and the overexpressing of it up-regulated expression of the H+/sugar antiporter genes, TST1 and TST2, by exporting glucose from vacuole to cytosol, which in turn led to increased glucose, fructose and sucrose in transgenic apple and tomato leaves and fruits (Zhu et al., 2021). In this study, we observed that overexpression of *MdSWEET23* in tomato could affect upstream sugar metabolic enzymes and the expression of sugar transporters. During the hexose accumulation phase in tomato, sugar unloading is the apoplastic pathway (Ruan and Patrick, 1995). Sucrose arrived in the SE/CC complex unloaded into the apoplasm by SUTs or catabolized to hexose by CWIN, and then hexose was transported to parenchyma cells by HTs (Zhang et al., 2021). In BC fruits of the *MdSWEET23*-OE tomato lines, more sucrose may unload into the apoplasm by *MdSWEET23*, leading to high CWIN activity to hydrolyze sucrose into hexoses(**Figure 1C**), which might be the cause of more accumulation of hexose in fruits of transgenic lines. Meanwhile, the expressions of hexoses transporter genes *HT1*, *HT2*, and *HT3* were also stimulated (**Figure 1D**)., although CWIN activity remained significantly higher as compared to WT. Ectopic overexpression of *MdSWEET23* may generate an unusual situation affecting the expression of sucrose transporter genes *SUT1*, *SUT2*, and *SUT4* (**Figure 1D**). Down-regulated *SUT1*, *SUT2*, and *SUT4* in RR fruits may negatively affect sucrose unloading, thereby influencing the enrichment of sucrose against the concentration gradient in fruits. Ectopic overexpression of *MdSWEET23* in tomato caused altered sugar metabolism and distribution but did not increase sugar sink potency in tomato fruits.

## Conclusions

This study demonstrates that *MdSWEET23*, derived from apples, functions as a sucrose transporter and is localized on the plasma membrane in vascular bundle tissues. It appears to participate in the unloading of sucrose in the phloem. Overexpression of this gene in apple calli significantly increased the sugar content. Conversely, when the gene was silenced during the phase of rapid sugar accumulation in the fruit, it negatively influenced sugar accumulation therein. The findings reveal that *MdSWEET23* possesses significant potential for enhancing the sugar content in fruit.

## Data availability statement

The original contributions presented in the study are included in the article/**Supplementary Files**, further inquiries can be directed to the corresponding authors.

## Author contributions

PN: Conceptualization, Data curation, Formal Analysis, Methodology, Project administration, Writing – original draft. LW: Data curation, Software, Formal Analysis, Validation, Writing – original draft. ML: Investigation, Methodology, Writing – review & editing, Data curation. DL: Investigation, Supervision, Writing – review & editing. SQ: Methodology, Project administration, Resources, Writing – review & editing. XX: Conceptualization, Funding acquisition, Writing – review & editing.

## References

[B1] Abelenda J. A. Bergonzi S. Oortwijn M. Sonnewald S. Du M. Visser R. G. F.. (2019). Source-sink regulation is mediated by interaction of an FT Homolog with a SWEET protein in potato. Curr. Biol. 29, 1178–1186.e6. doi: 10.1016/j.cub.2019.02.018 30905604

[B2] Breia R. Conde A. Badim H. Fortes A. M. Gerós H. Granell A. (2021). Plant SWEETs: from sugar transport to plant–pathogen interaction and more unexpected physiological roles. Plant Physiol. 186, 836–852. doi: 10.1093/plphys/kiab127 33724398 PMC8195505

[B3] Chardon F. Bedu M. Calenge F. Klemens P. A. Spinner L. Clement G.. (2013). Leaf fructose content is controlled by the vacuolar transporter SWEET17 in *Arabidopsis* . Curr. Biol. 23, 697–702. doi: 10.1016/j.cub.2013.03.021 23583552

[B5] Chen L. Q. (2014). SWEET sugar transporters for phloem transport and pathogen nutrition. New Phytol. 201, 1150–1155. doi: 10.1111/nph.12445 24649486

[B4] Chen H. Y. Huh J. H. Yu Y. C. Ho L. H. Chen L. Q. Tholl D.. (2015). The *Arabidopsis* vacuolar sugar transporter SWEET2 limits carbon sequestration from roots and restricts *Pythium* infection. Plant J. 83, 1046–1058. doi: 10.1111/tpj.12948 26234706

[B6] Chen L. Q. Lin I. W. Qu X. Q. Sosso D. McFarlane H. E. Londoño A.. (2015). A cascade of sequentially expressed sucrose transporters in the seed coat and endosperm provides nutrition for the *Arabidopsis* embryo. Plant Cell 27, 607–619. doi: 10.1105/tpc.114.134585 25794936 PMC4558658

[B7] Chen L. Q. Qu X. Q. Hou B. H. Sosso D. Osorio S. Fernie A. R.. (2012). Sucrose efflux mediated by SWEET proteins as a key step for phloem transport. Science 335, 207–211. doi: 10.1126/science.1213351 22157085

[B4000] Fan R. C. Peng C. C. Xu Y. H. Wang X. F. Li Y. Shang Y.. (2009). Apple sucrose transporter SUT1 and sorbitol transporter SOT6 interact with cytochrome b5 to regulate their affinity for substrate sugars. Plant Physiol. 150, 1880–1901. doi: 10.1104/pp.109.141374 19502355 PMC2719124

[B8] Geng Y. Q. Wu M. J. Zhang C. M. (2020). Sugar transporter ZjSWEET2.2 mediates sugar loading in leaves of *Ziziphus jujuba* Mill. Front. Plant Sci. 11. doi: 10.3389/fpls.2020.01081 PMC7396580 32849678

[B4001] Ho L. H. Klemens P. A. W. Neuhaus H. E. Ko H. Y. Hsieh S. Y. Guo W. J.. (2019). SlSWEET1a is involved in glucose import to young leaves in tomato plants. J. Exp. Bot. 70, 3241–3254. 30958535 10.1093/jxb/erz154 PMC6598072

[B9] Han L. Zhu Y. P. Liu M. Zhou Y. Lu G. Y. Lan L.. (2017). Molecular mechanism of substrate recognition and transport by the AtSWEET13 sugar transporter. Proc. Natl. Acad. Sci. U. S. A. 114, 10089–10094. doi: 10.1073/pnas.1709241114 28878024 PMC5617298

[B10] Hu L. P. Zhang F. Song S. H. Yu X. L. Ren Y. Zhao X. Z.. (2022). *CsSWEET2*, a hexose transporter from cucumber (*Cucumis sativus* L.), affects sugar metabolism and improves cold tolerance in *Arabidopsis* . Int. J. Mol. Sci. 23, 3886. doi: 10.3390/ijms23073886 35409244 PMC8999130

[B11] Jeena G. S. Kumar S. Shukla R. K. (2019). Structure, evolution and diverse physiological roles of SWEET sugar transporters in plants. Plant Mol. Biol. 100, 351–365. doi: 10.1007/s11103-019-00872-4 31030374

[B12] Jiang L. Song C. Zhu X. Yang J. (2021). SWEET transporters and the potential functions of these sequences in tea (Camellia sinensis). Front. Genet. 12. doi: 10.3389/fgene.2021.655843 PMC8044585 33868386

[B13] Kanno Y. Oikawa T. Chiba Y. Ishimaru Y. Shimizu T. Sano N.. (2016). AtSWEET13 and AtSWEET14 regulate gibberellin-mediated physiological processes. Nat. Commun. 7, 13245. doi: 10.1038/ncomms13245 27782132 PMC5095183

[B14] Kim P. Xue C. Y. Song H. D. Gao Y. Feng L. Li Y.. (2021). Tissue-specific activation of DOF11 promotes rice resistance to sheath blight disease and increases grain weight via activation of *SWEET14* . Plant Biotechnol. J. 19, 409–411. doi: 10.1111/pbi.13489 33047500 PMC7955873

[B15] Klemens P. A. W. Patzke K. Deitmer J. Spinner L. Le Hir R. L. Bellini C.. (2013). Overexpression of the vacuolar sugar carrier *AtSWEET16* modifies germination, growth, and stress tolerance in *Arabidopsis* . Plant Physiol. 163, 1338–1352. doi: 10.1104/pp.113.224972 24028846 PMC3813654

[B16] Kumar S. Stecher G. Li M. Knyaz C. Tamura K. (2018). MEGA X: Molecular evolutionary genetics analysis across computing platforms. Mol. Biol. Evol. 35, 1547–1549. doi: 10.1093/molbev/msy096 29722887 PMC5967553

[B17] Le Hir R. Spinner L. Klemens P. A. W. Chakraborti D. Marco F. Vilaine F.. (2015). Disruption of the sugar transporters AtSWEET11 and AtSWEET12 affects vascular development and freezing tolerance in *Arabidopsis* . Mol. Plant 8, 1687–1690. doi: 10.1016/j.molp.2015.08.007 26358680

[B18] Li X. Y. Guo W. Li J. C. Yue P. T. Bu H. D. Jiang J.. (2020). Histone acetylation at the promoter for the transcription factor PuWRKY31 affects sucrose accumulation in pear fruit. Plant Physiol. 182, 2035–2046. doi: 10.1104/pp.20.00002 32047049 PMC7140945

[B19] Li Y. Liu H. Yao X. Sun L. Sui X. (2022). The role of sugar transporter CsSWEET7a in apoplasmic phloem unloading in receptacle and nectary during cucumber anthesis. Front. Plant Sci. 12. doi: 10.3389/fpls.2021.758526 PMC8841823 35173746

[B20] Li Y. Liu H. Yao X. Wang J. Feng S. Sun L.. (2021). Hexose transporter CsSWEET7a in cucumber mediates phloem unloading in companion cells for fruit development. Plant Physiol. 186, 640–654. doi: 10.1093/plphys/kiab046 33604597 PMC8154047

[B21] Livak K. J. Schmittgen T. D. (2001). Analysis of relative gene expression data using real-time quantitative PCR and the 2(-Delta Delta C(T)) Method. Methods 25, 402–408. doi: 10.1006/meth.2001.1262 11846609

[B22] Loqué D. Lalonde S. Looger L. L. von Wirén N. Frommer W. B. (2007). A cytosolic *trans*-activation domain essential for ammonium uptake. Nature 446, 195–198. doi: 10.1038/nature05579 17293878

[B23] Lu C. Ye J. Chang Y. Mi Z. Liu S. Wang D.. (2022). Genome-wide identification and expression patterns of the *SWEET* gene family in *Bletilla striata* and its responses to low temperature and oxidative stress. Int. J. Mol. Sci. 23, 10057. doi: 10.3390/ijms231710057 36077463 PMC9456286

[B24] Ma S. Li Y. X. Li X. Sui X. L. Zhang Z. X. (2019). Phloem unloading strategies and mechanisms in crop fruits. J. Plant Growth Regul. 38, 494–500. doi: 10.1007/s00344-018-9864-1

[B25] Mathan J. Singh A. Ranjan A. (2021). Sucrose transport in response to drought and salt stress involves ABA-mediated induction of OsSWEET13 and OsSWEET15 in rice. Physiol. Plant 171, 620–637. doi: 10.1111/ppl.13210 32940908

[B26] Ni J. P. Li J. M. Zhu R. X. Zhang M. Y. Qi K. J. Zhang S. L.. (2020). Overexpression of sugar transporter gene *PbSWEET4* of pear causes sugar reduce and early senescence in leaves. Gene 743, 144582. doi: 10.1016/j.gene.2020.144582 32173543

[B27] Nie P. X. Wang X. Y. Hu L. P. Zhang H. Y. Zhang J. X. Zhang Z. X.. (2010). The predominance of the apoplasmic phloem-unloading pathway is interrupted by a symplasmic pathway during Chinese jujube fruit development. Plant Cell Physiol. 51, 1007–1018. doi: 10.1093/pcp/pcq054 20400534

[B28] Nie P. X. Xu G. X. Yu B. Lyu D. G. Xue X. M. Qin S. J. (2022). Genome-wide identification and expression profiling reveal the potential functions of the *SWEET* gene family during the sink organ development period in apple (*Malus × domestica* Borkh.). Agronomy 12, 1747. doi: 10.3390/agronomy12081747

[B29] Patrick J. W. (1997). Phloem unloading: sieve element unloading and post-sieve element transport. Annu. Rev. Plant Physiol. Plant Mol. Biol. 48, 191–222. doi: 10.1146/annurev.arplant.48.1.191 15012262

[B31] Ren Y. Sun H. Zong M. Guo S. Ren Z. Zhao J.. (2020). Localization shift of a sugar transporter contributes to phloem unloading in sweet watermelons. New Phytol. 227, 1858–1871. doi: 10.1111/nph.16659 32453446

[B32] Riesmeier J. W. Willmitzer L. Frommer W. B. (1992). Isolation and characterization of a sucrose carrier cDNA from spinach by functional expression in yeast. EMBO J. 11, 4705–4713. doi: 10.1002/j.1460-2075.1992.tb05575.x 1464305 PMC556945

[B33] Ruan Y. L. Patrick J. W. (1995). The cellular pathway of postphloem sugar transport in developing tomato fruit. Planta 196, 434–444. doi: 10.1007/BF00203641

[B34] Sonnewald U. (2001). Sugar sensing and regulation of photosynthetic carbon metabolism (The Netherlands: Kluwer Academic Publishers). doi: 10.1007/0-306-48148-0_6

[B35] Spolaore S. Trainotti L. Casadoro G. (2001). A simple protocol for transient gene expression in ripe fleshy fruit mediated by Agrobacterium. J. Exp. Bot. 52, 845–850. doi: 10.1093/jexbot/52.357.845 11413221

[B36] Sun H. J. Uchii S. Watanabe S. Ezura H. (2006). A highly efficient transformation protocol for Micro-Tom, a model cultivar for tomato functional genomics. Plant Cell Physiol. 47, 426–431. doi: 10.1093/pcp/pci251 16381658

[B41] Wang X. L. Xu Y. H. Peng C. C. Fan R. C. Gao X. Q. (2009). Ubiquitous distribution and different subcellular localization of sorbitol dehydrogenase in fruit and leaf of apple. J. Exp. Bot. 60, 1025–1034. doi: 10.1093/jxb/ern347 19174457 PMC2652060

[B38] Wang J. Xue X. Y. Zeng H. Q. Li J. K. Chen L. Q. (2022). Sucrose rather than GA transported by AtSWEET13 and AtSWEET14 supports pollen fitness at late anther development stages. New Phytol. 236, 525–537. doi: 10.1111/nph.18368 35811428 PMC9795879

[B37] Wang H. Yan S. Xin H. Huang W. Zhang H. Teng S.. (2019). A subsidiary cell-localized glucose transporter promotes stomatal conductance and photosynthesis. Plant Cell 31, 1328–1343. doi: 10.1105/tpc.18.00736 30996077 PMC6588317

[B39] Wang L. Yao L. Hao X. Li N. Qian W. Yue C.. (2018). Tea plant SWEET transporters: Expression profiling, sugar transport, and the involvement of CsSWEET16 in modifying cold tolerance in *Arabidopsis* . Plant Mol. Biol. 96, 577–592. doi: 10.1007/s11103-018-0716-y 29616437

[B42] Wang Y. F. Ye Z. Liu H. Liu Q. L. Zhang B. Hao Y. Y. (2015). Changes of vascular bundles structure and water transport of apple fruit in different development period. Plant Physiol. J. 51, 1414–1418. doi: 10.13592/j.cnki.ppj.2015.0367

[B40] Wang S. Yokosho K. Guo R. Whelan J. Ruan Y. L. Ma J. F.. (2019). The soybean sugar transporter GMSWEET15 mediates sucrose export from endosperm to early embryo. Plant Physiol. 180, 2133–2141. doi: 10.1104/pp.19.00641 31221732 PMC6670074

[B43] Wei X. Y. Liu F. L. Chen C. Ma F. W. Li M. J. (2014). The Malus domestica sugar transporter gene family: identifications based on genome and expression profiling related to the accumulation of fruit sugars. Front. Plant Sci. 5. doi: 10.3389/fpls.2014.00569 PMC4220645 25414708

[B44] Wieczorke R. Krampe S. Weierstall T. Freidel K. Hollenberg C. P. Boles E. (1999). Concurrent knock-out of at least 20 transporter genes is required to block uptake of hexoses in *Saccharomyces cerevisiae* . FEBS Lett. 464, 123–128. doi: 10.1016/S0014-5793(99)01698-1 10618490

[B4002] Yang J. Luo D. P. Yang B. Frommer W. B. Eom J. S.. (2018). SWEET11 and 15 as key players in seed filling in rice. New Phytol. 218, 604–615. 29393510 10.1111/nph.15004

[B45] Yao L. Ding C. Hao X. Zeng J. Yang Y. Wang X.. (2020). CsSWEET1a and CsSWEET17 mediate growth and freezing tolerance by promoting sugar transport across the plasma membrane. Plant Cell Physiol. 61, 1669–1682. doi: 10.1093/pcp/pcaa091 32645157

[B46] Zhang C. M. Bian Y. Hou S. H. Li X. G. (2018). Sugar transport played a more important role than sugar biosynthesis in fruit sugar accumulation during Chinese jujube domestication. Planta 248, 1187–1199. doi: 10.1007/s00425-018-2971-1 30094488

[B51] Zhang X. S. Feng C. Y. Wang M. N. Li T. L. Liu X. Jiang J. (2021). Plasma membrane-localized SlSWEET7a and SlSWEET14 regulate sugar transport and storage in tomato fruits. Hortic. Res. 8, 186. doi: 10.1038/s41438-021-00624-w 34333539 PMC8325691

[B48] Zhang L. Y. Peng Y. B. Pelleschi-Travier S. Fan Y. Lu Y. F. Lu Y. M.. (2004). Evidence for apoplasmic phloem unloading in developing apple fruit. Plant Physiol. 135, 574–586. doi: 10.1104/pp.103.036632 15122035 PMC429418

[B52] Zhang X. Y. Wang X. L. Wang X. F. Xia G. H. Pan Q. H. Fan R. C.. (2006). A shift of Phloem unloading from symplasmic to apoplasmic pathway is involved in developmental onset of ripening in grape berry. Plant Physiol. 142, 220–232. doi: 10.1104/pp.106.081430 16861573 PMC1557625

[B49] Zhang S. Wang H. Wang T. Zhang J. Liu W. Fang H.. (2023). Abscisic acid and regulation of the sugar transporter gene MdSWEET9b promote apple sugar accumulation. Plant Physiol. 192, 2081–2101. doi: 10.1093/plphys/kiad119 36815241 PMC10315282

[B50] Zhang W. Wang S. Yu F. Tang J. Shan X. Bao K.. (2019). Genome-wide characterization and expression profiling of SWEET genes in cabbage (*Brassica oleracea* var. capitata L.) reveal their roles in chilling and clubroot disease responses. BMC Genomics 20, 93. doi: 10.1186/s12864-019-5454-2 30696401 PMC6352454

[B47] Zhang H. P. Wu J. Y. Tao S. T. Wu T. Qi K.-j. Zhang S. J.. (2014). Evidence for apoplasmic phloem unloading in pear fruit. Plant Mol. Biol. Rep. Biol. Rep. 32, 931–939. doi: 10.1007/s11105-013-0696-7

[B53] Zhang Z. Zou L. M. Ren C. Ren F. R. Wang Y. Fan P.. (2019). VvSWEET10 mediates sugar accumulation in Grapes. Genes (Basel). 10, 255. doi: 10.3390/genes10040255 30925768 PMC6523336

[B55] Zhen Q. L. Fang T. Peng Q. Liao L. Zhao L. Owiti A.. (2018). Developing gene-tagged molecular markers for evaluation of genetic association of apple SWEET genes with fruit sugar accumulation. Hortic. Res. 5, 14. doi: 10.1038/s41438-018-0024-3 29581882 PMC5859117

[B54] Zhou Y. Liu L. Huang W. F. Yuan M. Zhou F. Li X. H.. (2014). Overexpression of OsSWEET5 in rice causes growth retardation and precocious senescence. PloS One 9, e94210. doi: 10.1371/journal.pone.0094210 24709840 PMC3978035

[B56] Zhu L. C. Li B. Y. Wu L. M. Li H. X. Wang Z. Y. Wei X. Y.. (2021). MdERDL6-mediated glucose efflux to the cytosol promotes sugar accumulation in the vacuole through up-regulating TSTs in apple and tomato. Proc. Natl. Acad. Sci. U. S. A. 118, e2022788118. doi: 10.1073/pnas.2022788118 33443220 PMC7817134

