# Supplementary material for: MdSWEET23, a sucrose transporter from apple (Malus × domestica Borkh.), influences sugar metabolism and enhances cold tolerance in tomato
Source: Front Plant Sci. 2023 Oct 3;14:1266194. doi: 10.3389/fpls.2023.1266194 (PMC10579938; doi:10.3389/fpls.2023.1266194)
Supplement: Supplementary file 1 [file DataSheet_1.pdf]

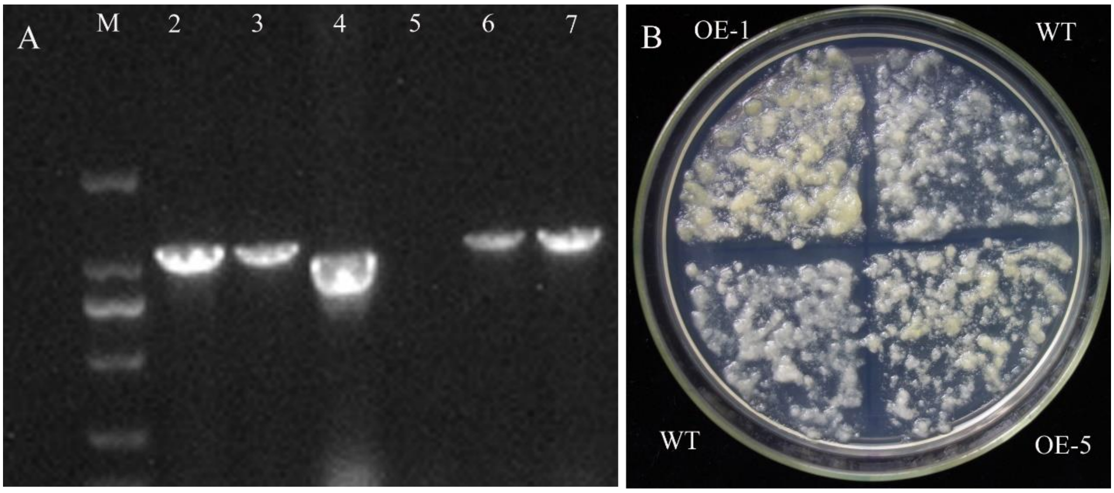

Fig. S1 Positive identification and growth of *MdSWEET23* over-expressing ‘’Orin’’ apple calli

Note: A shows the identification of ′′Orin″ apple calli positive strain, with lanes 1, 2, and 3–7 as DL2000 DNA marker, positive control, and transgenic detection of ‘Orin’ apple calli, respectively; B shows the growth situation of ′′Orin′′ calli after 2 weeks of culture.
