# Supplementary material for: MdSWEET23, a sucrose transporter from apple (Malus × domestica Borkh.), influences sugar metabolism and enhances cold tolerance in tomato
Source: Front Plant Sci. 2023 Oct 3;14:1266194. doi: 10.3389/fpls.2023.1266194 (PMC10579938; doi:10.3389/fpls.2023.1266194)
Supplement: Supplementary file 2 [file DataSheet_2.pdf]

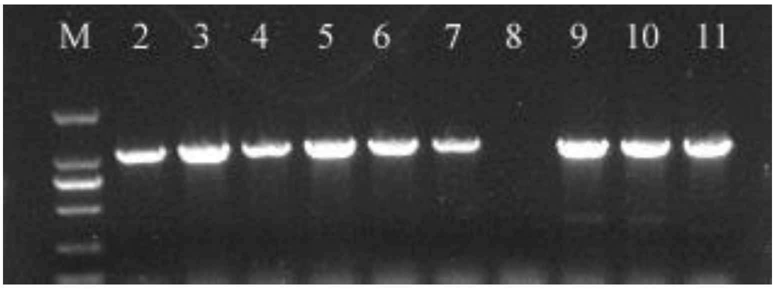

Fig. S2 Positive identification of *MdSWEET23* over-expressing tomato lines

Note: Lanes 1, 2, and 3–11 are DL2000 DNA markers, positive control, and tomato transgenic lines, respectively.
