# Supplementary material for: MdSWEET23, a sucrose transporter from apple (Malus × domestica Borkh.), influences sugar metabolism and enhances cold tolerance in tomato
Source: Front Plant Sci. 2023 Oct 3;14:1266194. doi: 10.3389/fpls.2023.1266194 (PMC10579938; doi:10.3389/fpls.2023.1266194)
Supplement: Supplementary file 3 [file Table_1.docx]

Table S1 Primers used in this study

| Assay | Primers name | Primer sequence (5′→3′) |
| --- | --- | --- |
| Gene expression | MdSWEET23-F | TCTTCTGTCTCACTTCTTGT |
|  | MdSWEET23-R | TGCGGATAACCATTCTCA |
|  | MdActin-F | TGACCGAATGAGCAAGGAAATTACT |
|  | MdActin-R | TACTCAGCTTTGGCAATCCACATC |
|  | MdSDH5-F | GCACTAGAGCCTGGTATC |
|  | MdSDH5-R | CTGGATGGACAACCTGAT |
|  | MdSDH6-F | TTCCGATACCAGAACACAT |
|  | MdSDH6-R | CACCTTGATGGCATTACC |
|  | MdSOT1-F | GGTTAGAATGACGTGGGCAGTTAT |
|  | MdSOT1-R | TCATCAACCTATTCACGGCCAC |
|  | pTRV2-test-F | CCACATATTCGCACGTATGA |
|  | pTRV2-test-R | GACTTACCGATCAATCAAGA |
|  | SlHT1-F | CGATGATCGAACGTGGTAAC |
|  | SlHT1-R | CAACAAGTTCCTCCAGGGAT |
|  | SlHT2-F | TCAACTACGGAACAGCCAAG |
|  | SlHT2-R | TCAGGTTCAATGTTGTCGGT |
|  | SlHT3-F | TGCGATAGCACAGTCTTTCC |
|  | SlHT3-R | CTTCGTTTCAGGCAAGAACA |
|  | SlSUT1-F | TTCCATAGCTGCTGGTGTTC |
|  | SlSUT1-R | TACCAGAAATGGGTCCACAA |
|  | SlSUT2-F | CCTACAGCGTCCCTTTCTCT |
|  | SlSUT2-R | GGATACAACCATCTGAGGTACAA |
|  | SlSUT4-F | TCTCCGCTGATATTGGATGG |
|  | SlSUT4-R | GCAACATCGAGAAGCCAAAA |
|  | SlACTIN-F | TGTCCCTATTTACGAGGGTTATGC |
|  | SlACTIN-R | AGTTAAATCACGACCAGCAAGAT |
|  | SlSWEET1a-F | TTTGCTGCTGTAGCCCTTGTT |
|  | SlSWEET1a-R | GAACTCCACGCTCTTCGTCTTG |
|  | SlSWEET3-F | TCCAAATCTGGTGGGAAC |
|  | SlSWEET3-R | TCAGTGAGAACGAGCATCG |
|  | SlSWEET2b-F | ATCTGGAACTCCGACGCAT |
|  | SlSWEET2b-R | AGGCACTCATTAGGAAGGTGG |
|  | SlSWEET3-F | TCCAAATCTGGTGGGAAC |
|  | SlSWEET3-R | TCAGTGAGAACGAGCATCG |
|  | SlSWEET2b-F | ATCTGGAACTCCGACGCAT |
|  | SlSWEET2b-R | AGGCACTCATTAGGAAGGTGG |
|  | SlSWEET10a-F | GAGTGAGAGACAAAGTGCTAGAAA |
|  | SlSWEET10a-R | AGCCCAATGACCAGAAATACC |
|  | SlSWEET10b-F | ACATTGTGAGGCTTGGTTTAATG |
|  | SlSWEET10b-R | TGCAACTTGGGCTCATTCT |
|  | SlSWEET11a-F | GAGATACAAAAGACTGAAGTGG |
|  | SlSWEET11a-R | CTACGTTTGTTTTATCAGTAAC |

Table S1 Primers used in this study (continued)

| Assay | Primers name | Primer sequence (5′→3′) |
| --- | --- | --- |
| Gene expression | SlSWEET11b-F | CAGGACTCACAGAGGAACAAA |
|  | SlSWEET11b-R | CATTGCGTCGTGCAAACA |
|  | SlSWEET12c-F | GCATCGTGTTTCAAGTGGTTCG |
|  | SlSWEET12c-R | TCTATCGCTGGCTTTGCGTT |
|  | SlSWEET14-F | TGAGTGCAATTGTGTGTTCAGA |
|  | SlSWEET14-R | GGCCTCTATGATTGCCTTTGG |
| gene clone | SWEET23-F | GTCGACATGTCTGCATCAACTTCTCACC |
|  | SWEET23-R | GGATCCTCAAACTTCACATGTAACAAGTTGG |
| vector construction | pDR196-SWEET23-F | TCGACTAGTGGATCCCCCGGGATGTCTGCATCAACTTCTCACCAT |
|  | pDR196-SWEET23-R | GATAAGCTTGATATCGAATTCTCAAACTTCACATGTAACAAGTTGGC |
|  | pBI121-*pMdSWEET23*-F | GACCATGATTACGCCAAGCTTTTTGACGACCCACTCTTGTAGGT |
|  | pBI121-*pMdSWEET23*-R | ACCACCCGGGGATCCTCTAGAGGTAGCTAGAGTTCTCTCTTTGGAGAT |
|  | pTRV2-MdSWEET23-F | TAAGGTTACCGAATTCACCAGAACACAAGGCTGATG |
|  | pTRV2-MdSWEET23-R | GCTCGGTACCGGATCCTGTAACAAGTTGGCTGGCATGG |

Table S2 The CDS sequence of *MdSWEET23* in ‘Hanfu’ apple

| ATGTCTGCATCAACTTCTCACCATCCATTGGCTTTTGCCTTTGGCATTCTAGGCAACATTGTCTCATTCATCGTTTTTCTAGCTCCGCTGCCGACATTTTGGAGGGTGTATAAAAAGAAATCGACGGAGGGATTTCAATCAGTTCCATATGTGTTTGCACTATTCAGTGCAATGATATGGATATACTATGCATTCCTCAAGTCTCATGTGATCCTGCTCATCACCATCAACTCATTCGGTTGTGTCATAGAGACCATTTATATTGCAATTTACCTTACATATGCAACTAAGCATGCGAGGGTGTCTACTCTGAGGCTGCTTTTACTGGTGAACTTCGGGGGATTTTGCTTGATTCTTCTTCTGTCTCACTTCTTGTCACAAGGGCCGACCCGCGTCGAAGTTCTTGGATGGGTTTGTGTGACTTTCTCTGTCAGTGTCTTTGCAGCACCTTTAAGCGTCATGAGAATGGTTATCCGCACCAAGAGCGTGGAGTTCATGCCGTTTAATTTATCCTTCTTCCTCACCCTAAGTGCCGTTATGTGGCTCTGTTATGGCTTACTCCTCAAGGATCTCTACGTTGCAACCCCAAACATACTTGGTTTTTCCTTTGGGGTGGTTCAGATGGCCCTCTATGCAAAGTACAGGAACACCAAAACAATTGTGGAGGAAAAGCTACCAGAACACAAGGCTGATGTTGTGAAGCAAATCAAAATTTTAACTACTACTCCTGAGGTGGAGGTACAAGTCCAAGCACCTGTAACCTCGGATACCAATAGTACTGATGCTCATCAAAACTCTGAGCGTGGCACTGACCAATATGTGCATGCACAAACATGCCGTAACGAGAAGATCATCGAACCATCCATGCCAGGCCAACTTGTTACATGTGAAGTTTGA |
| --- |

Note: Gray marked as VIGS fragment.

| Table S3 Characteristics of the SWEET23 in apple   \| Gene \| Protein physicochemical characteristics \| \| \| \| \| --- \| --- \| --- \| --- \| --- \| \|  \| Length (aa) \| MW (kDa) \| pI \| GRAVY \| \| MdSWEET23 \| 298 \| 33.48 \| 8.82 \| 0.497 \|   Table S4 Homology analysis between MdSWEET23 and AtSWEET1-17 | |
| --- | --- | --- | --- | --- | --- | --- | --- | --- | --- | --- | --- | --- | --- | --- | --- | --- |
|  | **MdSWEET23** |
| AtSWEET1 | 30.33% |
| AtSWEET2 | 28.38% |
| AtSWEET3 | 31.02% |
| AtSWEET4 | 27.81% |
| AtSWEET5 | 26.00% |
| AtSWEET6 | 26.49% |
| AtSWEET7 | 26.25% |
| AtSWEET8 | 25.74% |
| AtSWEET9 | 43.29% |
| AtSWEET10 | 42.95% |
| AtSWEET11 | 47.99% |
| AtSWEET12 | 48.99% |
| AtSWEET13 | 50.50% |
| AtSWEET14 | 49.50% |
| AtSWEET15 | 46.15% |
| AtSWEET16 | 26.76% |
| AtSWEET17 | 27.33% |
|  | |

Table S5 The sequence of promoter of *MdSWEET23*

| TTTGACGACCCACTCTTGTAGGTGATCACATGTTCTTCTCTCTTCTTAAAGAATGTGTTGGCTAAGAAGAGATCATATGCCATTGCAAAATCCAAGATAGCTTCCCCATCCTCGTTTCTCTCCCCAAAACCATGGCCACCATGAAAACCTCCATAGTTGCCTGTCTCCCTGCCCACGTGTCCATTTAAATCTCCTCCTATAAATAACTTCTCCGTCTGAGCAATTCCTTGCACCAAGTCTCCAAGGTCTTCCCAAAATTTCTCCTTCAAACTCATATCCAACCCTACTTGAGGTGCGTACGCACTAATCACATTGATAAGTTCTTGTCCTATTACAATCTTGATTGCCATGATTCTATCTCCTACCCTCTTGACATCTACAACATCTTGTGTCAAGGTCTTGTCCACGATGATGCCAACACCGTTTCTCGTTCTATTTGTGCCCGAATACCATAGTTTAAACCCTGAGTTTTCTAGATCCTTTGCCTTACGACCAACCCACTTAGTTTCTTGTAGGCACATAATATTTATCCTTCTCCTCACCATAACTTCTACTACTTCCATAGATTTTCCCGTCAAGGTTCCTATATTCCACGTTCCTAAACGCATTTTGCTCTCTTGAACTCTACCCTTCTGTCCTAGCTTCTTCACCCTTCCCCGTCTAATAGGATCAAAGTACTTCTTTTGTGTGTCCCGTGTAAAGTTGATAGGAGCATATGCTCCCAAACAACTTTGAGTGGAGTCGTTCGAAAAGAAGTTTCTATAACCCCCTTGCTCATTTAACATTGCATCCGGGTGCCGATGGAGATGCAGCGACCCTTGCTCACTTATCACTGTTCTCGGGCCACACAGCGCGCCACTTACGGGTGACGCCCTAGCTTTAGCGCGATTTCGTTCTGGATTCATTTTCATAAGGATTCGACGTGATCATGGAGTGCCGACTGTCGACTACCTGACGCCCTCCCCCTCCTCCTTTATCCGGGCTTGGGACCGGCAATGTAAGATAAACTTACACGGCAGAGTTTCATGATATGACATGTACATAATAAAATTTCATTCTCAACTCTACGATAGTTTTCATAAAAGTCTTTCATTTGCTTCTCTTTGATGCATGAAAGTTTTATTTACAAACAACATATCCTATTTTTTGTCATAATTCTTCTAGTGATCGATTCAACCTCTGATAAAGAAGATTATACGTACTTAGGTTATATTTCACCTCTTGAATAAGTTCAATAATGATCATAACAGCCCTATTGCTCCTATTGAGAGATGCGATCAATAAAGTAATCAGCAAGCGTGATAGAAGAAAATTTATTGAGAAGTTTCTGGGAATATTGAGAGGTTTTGGCGGTTGAATGAGCTTGAAAGTACTGCCAAAGCTAGCCAAAGAGCCGGAGTACTCCTAGAGAAACAGCTAAAAGCAATAGAAAGTGATAAACCGAAAAAGGAAGTGGCTTAGGAGGTGTCACGTGAAAGAGGAGCCAAGGAAGTTAACCTTTCCAAATATGTAACGTAAGTGTGTTGCACAATAATTAAAAATACCGACTCACATTTGTGAGTTGGCATTCACAAATATGCTTGTTCTAATGTCATGGGTGCTTGGCTTTTCTGTACTACAGCCACCTACTGCTAAACGCTAATAAAGTAATCATCACAATTCAGCCTTCATGCAACAACATAGCCTCATCTTCTCTCTATAAATACCAAGGACCCTTTGTATTCTGGATCTCGTGGCAAAGACGGAATATCAGAAAGATATAAGAGGATACAGGGCTAAGCTATATAGCTTCATAGTAAAACCCTGTTCCTTCAGTTTCAGTTTCAGTTGCTACACATCTCCAAAGAGAGAACTCTAGCTACC |
| --- |
